# Gene delivery in a mouse xenograft of a retargeted retrovirus to a solid 143B osteosarcoma

**DOI:** 10.1186/1743-422X-10-194

**Published:** 2013-06-14

**Authors:** Xia Zhang, Anindita Sarangi, Dai-Tze Wu, Jaya Kanduri, Monica J Roth

**Affiliations:** 1 Department of Pharmacology, University of Medicine and Dentistry of New Jersey–Robert Wood Johnson Medical School, 675 Hoes Lane, Piscataway, NJ, 08854, USA; 2 Department of Biochemistry, University of Medicine and Dentistry of New Jersey–Robert Wood Johnson Medical School, 675 Hoes Lane, Piscataway, NJ, 08854, USA

**Keywords:** 143B osteosarcoma, Targeted retroviral entry, FeLV Env, MLV luciferase delivery

## Abstract

**Background:**

Osteosarcomas are the most common primary bone malignancies found in children and adolescents. An optimized system was developed for efficient retroviral gene delivery into solid 143B osteosarcoma tumors in mice using a retargeted Env. In these studies, the viral Env CP was isolated from an in vitro screen of a library of feline leukemia virus Env randomized in the receptor-binding domain and maintained high titer on human 143B osteosarcoma cell line.

**Findings:**

The vector developed to express the random Env libraries encoded the drug selectable marker *neo*. To adapt this for studies in live animals, the murine based vector was modified to express the *luciferase* gene. The bicistronic vector developed expressed both the CP Env and luciferase in the presence of either the MPMV CTE or a WPRE element. Virus bearing the CP FeLV Env variant maintained high titers after concentration allowing for direct visualization of delivery of the luciferase gene in subcutaneous 143B osteosarcoma tumors.

**Conclusion:**

This system serves as a proof-of-concept for the use of novel FeLV Env pseudotyped MLV particles for in vivo gene delivery. Gene delivery and expression of lucerifase from viral particles bearing the CP Env was readily detected in live mice after a single round of intratumor injection.

## Findings

For children and adolescents, osteosarcomas are the most common primary bone malignancies (for review [1]). Micrometastasis frequently occurs to the lung, with detection difficult at the time of primary diagnosis [2,3]. Even after resection and chemotherapy treatment [4-6], approximately 30% of patients, in particular those with metastasis, remain with a poor prognosis [6]. Studies have identified genes which specifically suppress in vitro migration and invasion of osteosarcomas, including LRP5 [7] and IGFBP5 [8]. Thus, the ability to deliver genes or RNA species that can inhibit tumor growth and metastasis is of interest.

A long-term interest in improving the safety of retroviral vector usage is to identify Env proteins that can specifically target a cell type of interest. A key limitation of in vivo retroviral gene delivery is the overall specificity and titer of the particle delivered. Novel retargeted viruses frequently maintain low titer, and are thus not suitable for animal studies [9]. Our laboratory has developed a screen to identify novel retroviral Env capable of productively infecting target cells through randomizing an 11 amino acid region of the receptor binding domain of feline leukemia virus (FeLV) [10-12]. Through screening retroviral Env libraries, isolates including CP and L1, capable of infecting the human osteosarcoma cell line 143B were identified [11,12]. To establish the effectiveness of intratumor delivery, studies were initiated with the CP isolate, which maintained high titers in 143B cells in vitro and was stable after concentration [11,12]. The cognate cellular receptors for CP have been identified (SLC52A1, SLC52A2) as the putative riboflavin transporters, with a permissive host range including human but not porcine, murine or lapine cells [12,13].

The current report examines the ability of viral particles pseudotyped with a retargeted viral Env to deliver transgenes to xenograft tumors of human osteosarcoma cells in an athymic mouse. With the goal of adapting the CP Env system to live animal studies, the pRVL vector was modified to replace the *neo* gene with *luciferase,* a marker suitable for non-invasive imaging in mice [14]. Details on the construction of the vectors and the oligonucleotides sequences are provided in the Additional file 1.

293TCeB cells [10], which stably express Mo-MLV *gag* and *pol* gene from the CeB plasmid, were maintained in DMEM (Gibco) supplemented with 10% fetal bovine serum (Atlanta Biologicals), 10 μg/ml blasticidin (InvivoGen) and antibiotic-antimycotic (Gibco). To assemble viral particles, 1 × 10^6^ 293TCeB cells were transfected with 2.5 μg of each plasmid construct with Lipofectamine 2000 (Invitrogen) at 37°C for 6 h. After 24 h, cells were treated with 10 mM sodium butyrate at 37°C for 5 h. Supernatants were collected after 48 h, filtered through a 0.45 μm filter and used to infect 2 × 10^5^ 143B cells. For each construct, three independent transfections were performed. Three days post-infection, 143B cells were lysed with 150 μl of lysis reagent; 20 μl of cell lysate was mixed well with 100 μl of luciferase assay reagent and the relative light units (RLU) of luciferase activity was determined using the Luciferase assay system kit (Promega). Luciferase activity was performed on 20/20 luminometer (Promega), programmed with a 2-second measurement delay followed by a 10-second reading for the enzyme activity.

Initial vectors examined the expression of the *luciferase* gene driven by an internal SV40 promoter, the EF1α promoter, or an IRES element. Use of either the internal SV40 or EF1α promoter resulted in aberrant splicing into the internal promoter regions, eliminating the packaging of the RNA into viral particles (data not shown).

**Figure 1:**
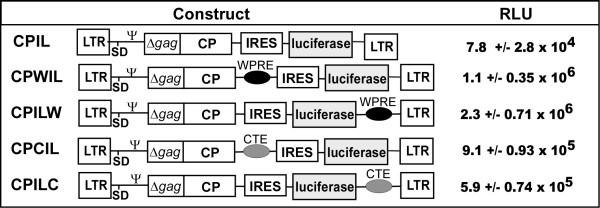
**Gene transduction of luciferase from murine retroviral based vectors.** Schematic of the panel of MLV based vectors expressing luciferase and their transduction activity. Effects of CTE and WPRE elements on the gene transfer of luciferase within a CPIL backbone. LTR, long terminal repeat; ψ, RNA packaging region; SD, splice donor; SA, splice acceptor. RLU, relative light units of luciferse activity. Assays were performed in triplicate and the results are shown as mean ± standard deviation. P-values from one tailed student t-tests (N = 3); comparing CPILW individually to CPIL, CPWIL, CPCIL or CPILC were all p < 0.001.

Maximal luciferase transfer was observed with vectors expressing the luciferase gene from the IRES elements (CPIL vector) (Figure 1). Two genetic modulators, WPRE [15,16] and CTE [17], were incorporated into CPIL vector, positioned 3’ of either the *env* or the *luc* genes (Figure 1). The presence of either of the elements greatly increased the transfer of luciferase into target cells with small but statistically significant positional variation. Maximal luciferase transfer was observed with vector CPILW, showing a 29-fold increase in activity (2.3 ± 0.71 × 10^6^ RLU). RT-PCR of RNA extracted from 293TCeB cell transfected with the CPILW construct readily detected the unspliced vRNA; the presence of a cryptic splice site within p12 [18] was also identified (data not shown).

A key interest is to define the efficiency of gene transfer into mouse tumor models. The ability of retroviral particles bearing CP Env to deliver genes to solid tumors was examined in athymic mice (female, athymic Nude-*Foxn1*^*n*^*u, 6–8 weeks, Harlan Laboratories*). 10^6^ 143B cells were suspended in 100 μl PBS and injected subcutaneously, close to the armpit. Injection was performed on both sides of each mouse. Fresh viral particles were assembled in 293TCeB cells in the presence of either CPILW, dEnvILW or VSV-G as described above. 10 ml of filtered viral supernatant was precipitated with 10% PEG 8000 at 4°C overnight, pelleted at 1847× g for 45 minutes and resuspended in 100 μl PBS (~50 μg of MLV p30). The amount of each virus used for injection was normalized to the amount of MLV p30 detected by ELISA [19]. When tumors grew to ~0.5 cm in diameter (~ 5 days), a single intratumor injection of viral particles (100 μl) was performed at one side of the animal. Three days after intratumoral viral injection, mice were anesthetized by isoflurane inhalation and injected intraperitoneally with 100 μl of Redirect D-luciferin Ultra (concentration of 30 mg/ml; Caliper LifeScience). Bioluminescence recordings were taken by IVIS 200 Series System (Caliper LifeScience). The image acquisition time was 5 minutes. Post-processing and quantification was performed using Living Image Software (Xenogen).

**Figure 2:**
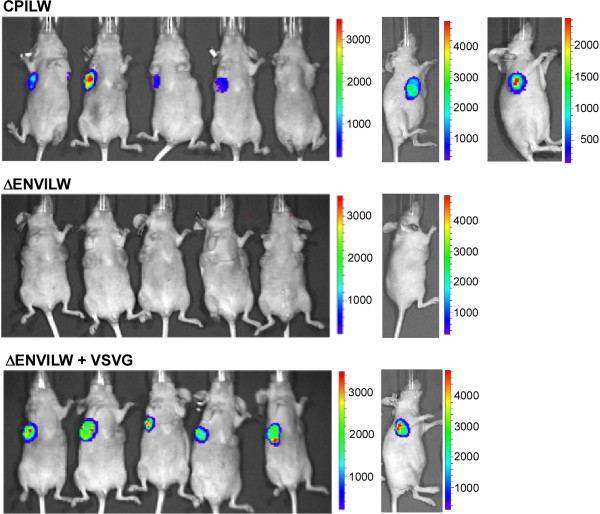
**Target gene transfer in tumor models.** Bioluminescence recordings of athymic mice injected intratumorally with viral particles and analyzed in the presence of 3 mg of Redirect D-luciferin Ultr. Viral constructs are as labeled.

Luciferase gene transfer by retroviral particles in the subcutaneous 143B osteosarcoma tumors was examined. Initial experiments in 143B cells indicated that a single round of delivery was sufficient for detection (Figure 2). Three sets of viral particles were studied (Figure 2). The experimental system contained the CPILW vector, expressing both the CP Env and the luciferase marker. ∆EnvILW served as the negative control, lacking the CP Env and maintaining the *luc* gene. ∆EnvILW pseudotyped with VSV-G allowed for entry through an alternative viral Env. Viral particles bearing the CP Env expressed luciferase in the tumors at the site of injection consistent with the boundaries of the tumors, with the signal intensity varying among individuals. In the mice injected with CP-pseudotyped viral particles, 86% (6/7) displayed signals. Tumors from the animal that did not show luciferase activity had signs of necrosis. No luciferase was detected in the 143B tumors on the opposite side of the animal or in any other mouse tissue (N = 7). Evidence of metastasis of the 143B cells to other tissue [20] was not visually observed.

As a positive control, all of the mice injected with VSV-G-pseudotyped luciferase-containing virus displayed significant signals (N = 6), whereas no signals were detected among the mice injected with Env-free ∆EnvILW viral particles (N = 6), indicating that the delivery of viral content was Envelope dependent and not through a non-specific endocytic mechanism.

Structurally and biochemically, the VSV-G Env is distinct from that of the FeLV Env [21]. It is a homo-trimer of a single-transmembrane pass protein whose receptor, albeit unknown, is ubiquitously expressed in both vertebrates and insect cells. FeLV Env are trimers of heterodimers, where the subunit responsible for binding the host-cell receptor is not associated with the viral membrane and therefore is susceptible to shedding from the particles. Therefore, it was not anticipated that in live animals, the delivery of virus bearing the CP Env through binding to its cognate receptor, would be equivalent to that of the non-specific VSV-G protein. These studies indicate that novel Env proteins selected by library screening were stable for high titer intratumor delivery into solid tumors. Significantly, luciferase expression could be directly observed after a single injection of viral particles.

For single-round of transgene delivery, a bicistronic vector is not required. However in the athymic mice, if virus packaging the CPILW vector infect 143B cells that stably express the MLV *gag* and *pol* genes (143B/CeB [11]), additional rounds of transduction would be possible. This amplification system serves as an alternative to the use of replication competent virus [22]. Initial studies indicate that expression of *gag/pol* genes did result in signal amplication (data not shown). This approach can facilitate future studies of micrometastasis, where the migration of tumor cells can be identified by gene delivery of luciferase to secondary sites by the released CP Env virus. Future experiments will require monitoring the 143B cells and the viral particles with separate visual markers. To this end, the 143B cells are currently being labeled with renilla *luciferase*.

These studies validate the effectiveness of this viral delivery system in the 143B osteosarcoma animal model. Although CP Env maintains high titers on 143B cells, its tissue specificity is not limited to osteosarcoma cells [12,13]. Additional specificity for in vivo targeted gene delivery to osteosarcomas could be obtained using the FeLV L1 Env isolate [11], which appears to have a more limited host and tissue tropism.

Animal studies were performed following the approved UMDNJ-RWJMS protocol # I12-026-5.

## Competing interests

The authors declare that they have no competing interest.

## Authors’ contributions

XZ constructed the vectors, performed the animal studies and assisting in writing the manuscript. AS and JK assisted in generating the vectors. D-TWu assisted in the animal studies and virus preparation. MJR designed the experiments, analyzed the data, and wrote the manuscript. All authors approved the final manuscript.

## Supplementary Material

Additional file 1 Additional text and table. Click here for file
