# Supplementary material for: Gene delivery in a mouse xenograft of a retargeted retrovirus to a solid 143B osteosarcoma
Source: Virol J. 2013 Jun 14;10:194. doi: 10.1186/1743-422X-10-194 (PMC3689073; doi:10.1186/1743-422X-10-194)
Supplement: Additional file 1 — Additional text and table. [file 1743-422X-10-194-S1.pdf]

Additional Text and Table

Materials and methods.

Plasmids. pGL3 (luciferase gene) was obtained from Promega, pGIP [1] and HIV-2gtPGIPW from Dr. J. Dougherty (UMDNJ), pSNVU3RU5-CTE from Dr. Boris-Lawrie (Ohio State University).

Plasmid constructs. The parental plasmid pSL was generated by replacing the *neo* gene from pRVL [2] with the luciferase cassette. pRVL was digested with BamHI, removing the Env cassette (pRVL ΔEnv template). Sequences of individual primers are in Table 1. Sequential overlapping PCR between products of primers 1F/1R (400 bp, pRVL ΔEnv template), 2F/2R (1680 bp, Luciferase, pGL3 template (Promega)), and 3F/3R (~800 bp, pRVL template) yielded a ~2800 bp product that was cloned into pRVL ΔEnv template using the BamHI and ClaI restriction sites, generating pSL. pIL substituted the SV40 promoter with the IRES element derived from pGIP [1]. Overlapping PCR fragment were assembled using primers 6F/6R on pGIP and 7F/7R on pRVL ΔEnv template and cloned into pSL using the BamHI/BstBI sites. The CP env gene was introduced into pIL vector, generating CPIL through ligation of the 2-kb BamHI CP env cassette into BamHI-linearized pIL and the orientation was confirmed by sequencing.

Insertion of WPRE and CTE elements. Plasmid HIV-2gtPGIPW encodes the WPRE element [3] flanked by BamHI and BglII sites. The WPRE was modified, mutating the ATG of the oncogenic X-protein to TTG and the upstream sequence AAGCTGT to AAATCAT as described in ([4], mut6). The WPRE sequence was amplified from HIV-2gtPGIPW with primers 12F/12R, the 620 WPRE fragment was digested with BamHI and BglII and inserted into BamHI digested pCPIL, generating pWIL. The BamHI-BamHI CP Env cassette was subsequently reintroduced into the plasmid generating pCPWIL and the orientation of the Env cassette was verified by DNA sequencing. The CTE was amplified from plasmid SNVU3RU5-CTE, using primers 16F/16R. The 170 bp CTE fragment was digested with BamHI and BglII and similarly ligated into BamHI digested pCPIL, generating pCIL, into which the BamHI-BamHI CP Env cassette was subsequently reintroduced (pCPCIL). The pCPILW construct was assembled through overlapping PCR of fragments encoding *luc* (pGL3 as template and primers 13F/13R), WPRE (HIV-2gtPGIPW as template and primers 14F/14R) and the LTR (pIL as template and primers 15F/15R). The 1.3 kB fragment was digest with BstBI and ClaI, and exchange into CPIL, forming CPILW. ΔEnvILW was generated by removing the BamHI-BamHI Env cassette. The CPILC construct was assembled through overlapping PCR of fragments encoding Luc (pGL3 as template and primers 17F/17R), CTE (SNVU3RU5 as template and primers 18F/18R) and the LTR (pIL as template and primers 19F/19R), digested with BstBI and ClaI, and exchanged into CPIL, forming CPILC.

References

1. Chen C-C, Rivera A, Ron N, Dougherty JP, Ron Y: **A gene therapy approach for treating T-cell-mediated autoimmune diseases.** *Blood* 2001, **97:**886-894.
2. Bupp K, Roth MJ: **Altering retroviral tropism using a random-display envelope library.** *Mol Ther* 2002, **5:**329-335.
3. Donello JE, Loeb JE, Hope TJ: **Woodchuck hepatitis virus contains a tripartite posttranscriptional regulatory element.** *J Virol* 1998, **72:**5085-5092.
4. Zanta-Boussif MA, Charrier S, Brice-Ouzet A, Martin S, Opolon P, Thrasher AJ, Hope TJ, Galy A: **Validation of a mutated PRE sequence allowing high and sustained transgene expression while abrogating WHV-X protein synthesis: application to the gene therapy of WAS.** *Gene Therapy* 2009, **16:**605-619.

Table 1: Summary of Primers and Templates for Generation of Luciferase Retroviral Vectors

	Forward primer[1]	Reverse primer[1]	Template	Construct
1	5'-tccagccctcactccttctctag-3'	5'-gtttttggcgtcttccatgcgaaacgatcctca-3'	pRVL	pSL
2	5'-atggaagacgccaaaaacataaa-3'	5'-ttacacggcgatctttccgccc-3'	pGL3	
3	5'-gaaagatcgccgtgtaagcgggactctggggt-3'	5'-attccagaagtagtgaggagg-3'	pRVL	
6	5'-gccggatccgaattccgcccctctcct-3'	5'-gttgtggccatataatcatcgtgttt-3'	pGIP	pIL
7	5'- tatggccacaacgccaccatggaagacgcc-3'	5'-gtgatttgtattcagccca-3'	pRVL ΔEnv	
12	5'-cctgtaggtttggcaagc-3'	5'-tccatgccttgcaaaatgg-3'	HIV-2gtPGIPW	pCPWIL
13	5'-gaggtggacatcacttacg-3'	5'-gaggttgattaccggtttacacggcgatct-3'	pGL3	pCPILW
14	5'-gtaaaccggtaatcaacctctggattac-3'	5'-gagtcccgccaggcggggaggcggc-3'	HIV-2gtPGIPW	
15	5'-ccgcctggcgggactctggggt-3'	5'-aatccagaagtagtgaggacg-3'	pIL	
16	5'-gaaggatccagactggacagccaa-3'	5'-gctagatctaattataaaaaacaaaag-3'	pSNVU3RU5-CTE	pCPCIL
17	5'-gaggtggacatcacttacg-3'	5'-tgtccagtctaccggtttacacggcgatct-3'	pGL3	pCPILC
18	5'-gtaaaccggtagactggacagccaatg-3'	5'-gagtcccgcaattataaaaaacaa-3'	pSNUU3RU5	
19	5'-tataattgcgggactctggggtc-3'	5'-aatccagaagtagtgaggacg-3'	pIL	

[1]Sequences underlined highlight restriction sites introduced in the primers.
